# Different aspects of early and late development of atrial fibrillation during hospitalization in cryptogenic stroke

**DOI:** 10.1038/s41598-021-86620-5

**Published:** 2021-03-29

**Authors:** Ryosuke Doijiri, Yuji Ueno, Muneaki Kikuno, Takahiro Shimizu, Yohei Tateishi, Ayako Kuriki, Hidehiro Takekawa, Yoshiaki Shimada, Kodai Kanemaru, Yuki Kamiya, Eriko Yamaguchi, Masatoshi Koga, Masafumi Ihara, Akira Tsujino, Koichi Hirata, Yasuhiro Hasegawa, Takahiko Kikuchi, Nobutaka Hattori, Takao Urabe

**Affiliations:** 1 Department of Neurology, Iwate Prefectural Central Hospital, Iwate, Japan; 2 Department of Neurology, Juntendo University School of Medicine, Tokyo, Japan; 3 Department of Cerebrovascular Medicine, National Cerebral and Cardiovascular Center, Osaka, Japan; 4 Department of Neurology, Tokyo Medical School, Tokyo, Japan; 5 Department of Neurology, St. Marianna University School of Medicine, Kanagawa, Japan; 6 Department of Neurology and Strokology, Nagasaki University Hospital, Nagasaki, Japan; 7 Department of Neurology, Showa University Koto Toyosu Hospital, Tokyo, Japan; 8 Department of Neurology, Dokkyo Medical University, Tochigi, Japan; 9 Department of Neurology, Juntendo University Urayasu Hospital, Chiba, Japan; 10 Department of Neurology, National Cerebral and Cardiovascular Center, Osaka, Japan

**Keywords:** Cerebrovascular disorders, Stroke

## Abstract

The detection of underlying atrial fibrillation (AF) has become increasingly possible by insertable cardiac monitoring (ICM). During hospitalization for cryptogenic stroke, factors related to the early and late development of AF have not been studied. CHALLENGE ESUS/CS is a multicenter registry of cryptogenic stroke patients undergoing transesophageal echocardiography. Twelve-lead electrocardiogram, continuous cardiac monitoring, and 24-h Holter electrocardiogram were all used for the detection of AF. Early and late detection of AF was determined with an allocation ratio of 1:1 among patients with AF. A total of 677 patients (68.7 ± 12.8 years; 455 men) were enrolled, and 64 patients developed AF during hospitalization. Four days after admission was identified as the approximate median day to classify early and late phases to detect AF: ≤ 4 days, 37 patients; > 4 days, 27 patients. Multiple logistic regression analysis showed that spontaneous echo contrast (SEC) (OR 5.91; 95% CI 2.19–15.97; *p* < 0.001) was associated with AF ≤ 4 days, whereas a large infarction > 3 cm in diameter (OR 3.28; 95% CI 1.35–7.97; *p* = 0.009) was associated with AF > 4 days. SEC and large infarctions were important predictors of in-hospital AF detection, particularly in the early and late stages, respectively; thus, they could serve as indications for recommending ICM.

## Introduction

Cryptogenic stroke, known as ischemic stroke with an undermined etiology, is not uncommon and comprises about a fifth to a quarter of all ischemic strokes^1^. The majority of cryptogenic strokes are caused by embolic mechanisms; thus, an embolic stroke of undetermined source (ESUS) was advocated in 2014^1,2^. Underlying atrial fibrillation (AF) is a primary embolic source in cryptogenic stroke and ESUS among various potential embolic diseases, including arteriogenic embolisms and paradoxical embolisms^3,4^. Short-term monitoring, such as continuous inpatient telemetry and Holter monitoring, has been shown to detect approximately 8% of covert AF cases in cryptogenic stroke patients; meanwhile, detection of AF increased to approximately 30% with extended cardiac monitoring, such as insertable cardiac monitoring (ICM), during 3 years of follow-up^5–7^. In recent clinical trials, direct oral anticoagulants did not reduce the risk of stroke in comparison to aspirin among individuals with ESUS^8,9^. Thus, determining the optimal anticoagulant therapy is essential to elucidate the clinical manifestations of AF among embolic diversities in cryptogenic stroke and ESUS.

Recent studies have focused on the clinical significance of the AF development stage^10–12^. Late-onset of AF during an acute myocardial infarction increased the risk of stroke^11^. After catheter ablation, early- and late-phase AF recurrence displayed different clinical characteristics^10,12^. Furthermore, although considerable evidence has found that extended cardiac monitoring, such as ICM, substantially increased AF identification, the related clinical characteristics are yet to be elucidated. Despite these interests, there is no available evidence regarding the impact of early- and late-phase AF detection, and for clarifying the clinical manifestations of patients with late-phase AF detection to enable stratifying patients for whom prolonged cardiac monitoring, such as ICM, is indicated in cryptogenic stroke.

Transesophageal echocardiography (TEE) is critical to assess cardiac parameters related to AF, screen for diverse potential embolic sources in cryptogenic stroke and ESUS, and guide optimal therapeutic management^3,13^. The presence of a thrombus, spontaneous echo contrast (SEC), and decreased left atrial appendage (LAA) emptying velocity are reported markers of thromboembolic risk in AF^14–16^. We have recently initiated the Mechanisms of Embolic Stroke Clarified by Transesophageal Echocardiography for ESUS/Cryptogenic Stroke (CHALLENGE ESUS/CS) registry, enrolling cryptogenic stroke patients undergoing TEE as well as maintaining a comprehensive database among multiple centers in Japan. Using data from CHALLENGE ESUS/CS registry with comprehensive data including detection of AF, we explored the clinical characteristics of patients with early and late development of AF in the hospital after cryptogenic stroke.

## Methods

The CHALLENGE ESUS/CS registry is a multicenter, retrospective registry that enrolled consecutive patients with cryptogenic stroke who underwent TEE within eight participating hospitals in Japan between April 2014 and December 2016, and was registered at http://www.umin.ac.jp/ctr/ (UMIN000032957). Details of the rationale, study design, factors to evaluate, TEE protocol, and characteristics of the participants in the CHALLENGE ESUS/CS registry have been published elsewhere^17,18^. We used clinical information obtained from medical records, and therefore, the need to obtain written informed consent from each patient was waived for this retrospective study. The Independent Ethics Committee of Juntendo University Hospital, the Independent Ethics Committee of Iwate Prefectural Central Hospital, the Research Ethics Committee of the National Cerebral and Cardiovascular Center, the Ethics Committee of St. Marianna University Hospital, the Nagasaki University Hospital Clinical Research Ethics Committee, the Ethics Committee of Showa University Koto Toyosu Hospital, the Independent Ethics Committee of Dokkyo Medical University Hospital, and the Independent Ethics Committee of Juntendo University Urayasu Hospital all approved the protocol. This study was conducted following the Declaration of Helsinki.

### Inclusion and exclusion criteria for the CHALLENGE ESUS/CS registry

Inclusion criteria for the current study were as follows: (1) ischemic stroke within seven days of onset; (2) non-lacunar infarction on neuroradiological imaging; (3) absence of an arterial stenosis > 50% or occlusive lesions in a corresponding cervical or large cerebral artery; (4) absence of major cardiac embolic sources; and (5) lack of other determined stroke etiologies. In the diagnostic criteria of ESUS, cardiac monitoring for > 24 h is recommended; thus, AF that was detected < 24 h after admission was not suitable for the diagnosis of ESUS and was not registered in the CHALLENGE ESUS/CS registry.

### Atherosclerotic risk factors

Hypertension was defined as a history of using antihypertensive agents, systolic blood pressure > 140 mmHg, or diastolic blood pressure > 90 mmHg at 14 days after stroke. Diabetes mellitus was defined as the use of oral hypoglycemic agents or insulin, fasting blood glucose level > 126 mg/dL, or glycosylated hemoglobin ≥ 6.5%. Dyslipidemia was defined as use of antihyperlipidemic agents, serum low-density lipoprotein cholesterol ≥ 140 mg/dL, high-density lipoprotein cholesterol < 40 mg/dL, or triglycerides ≥ 150 mg/dL. Chronic kidney disease (CKD) was defined as estimated glomerular filtration rate < 60 mL/min/1.73 m^2^.

### TEE

TEE was performed as explained in our previous work^4,13^. Subjects were awake and had fasted for at least 4 h before the examination. To examine the heart and aortic arch, a multiplane probe was manipulated to provide appropriate views, including axial and sagittal images. The presence of a right-to-left shunt was assessed by injecting agitated saline and having patients perform the Valsalva maneuver, and then numbers of microbubbles with and without contrast agents were compared. Atrial septal aneurysm was defined as a ≥ 10 mm excursion into either the left or right atrium, or a sum of total excursion into the left or right atrium of ≥ 15 mm. Aortic arch plaques with thickness ≥ 4 mm, mobile plaques seen swinging on their peduncles, or ulcerative plaques with width and maximum depth of at least 2 mm were defined as aortic complicated lesions. Examinations were performed by two or three experienced sonographers in each institution.

### In-hospital detection of AF and temporal classification

After admission, all enrolled patients underwent a 12-lead electrocardiogram, continuous cardiac monitoring, and a 24-h Holter electrocardiogram after admission. To date, evidence regarding classification into the early to late stages of in-hospital AF detection has been unavailable. The cutoff period to classify early and late detection of AF was determined by a ratio approximating 1:1 allocation.

### Data collection and analyses

Collection of baseline clinical information including cardiovascular risk factors, laboratory and radiological data on admission, and echocardiographic findings was conducted with permission by the steering committee of the CHALLENGE ESUS/CS. These variables were compared according to the absence of AF, early detection of AF, and late detection of AF. Independent factors associated were assessed in those with early and late AF detections.

### Statistical analysis

Numerical values are reported as the mean ± standard deviation. Data were analyzed using the chi-squared test for categorical variables and the Kruskal–Wallis test for nonparametric analyses among patients with early detection of AF after admission, late detection of AF after admission, and no AF. All variables with values of *p* < 0.05 on univariate analyses were entered into a multinomial regression analysis to identify independent variables for patients with the development of AF in the early and late phases. Predictors for in-hospital AF detection were analyzed using the Kaplan–Meier analysis. A value of *p* < 0.05 was considered statistically significant. All data analyses were conducted using SPSS for Mac version 26.0 (SPSS Inc., Chicago, IL, USA).

### Data availability

The datasets used and analyzed during the current study are available from the corresponding author upon reasonable request.

## Results

**Figure 1:**
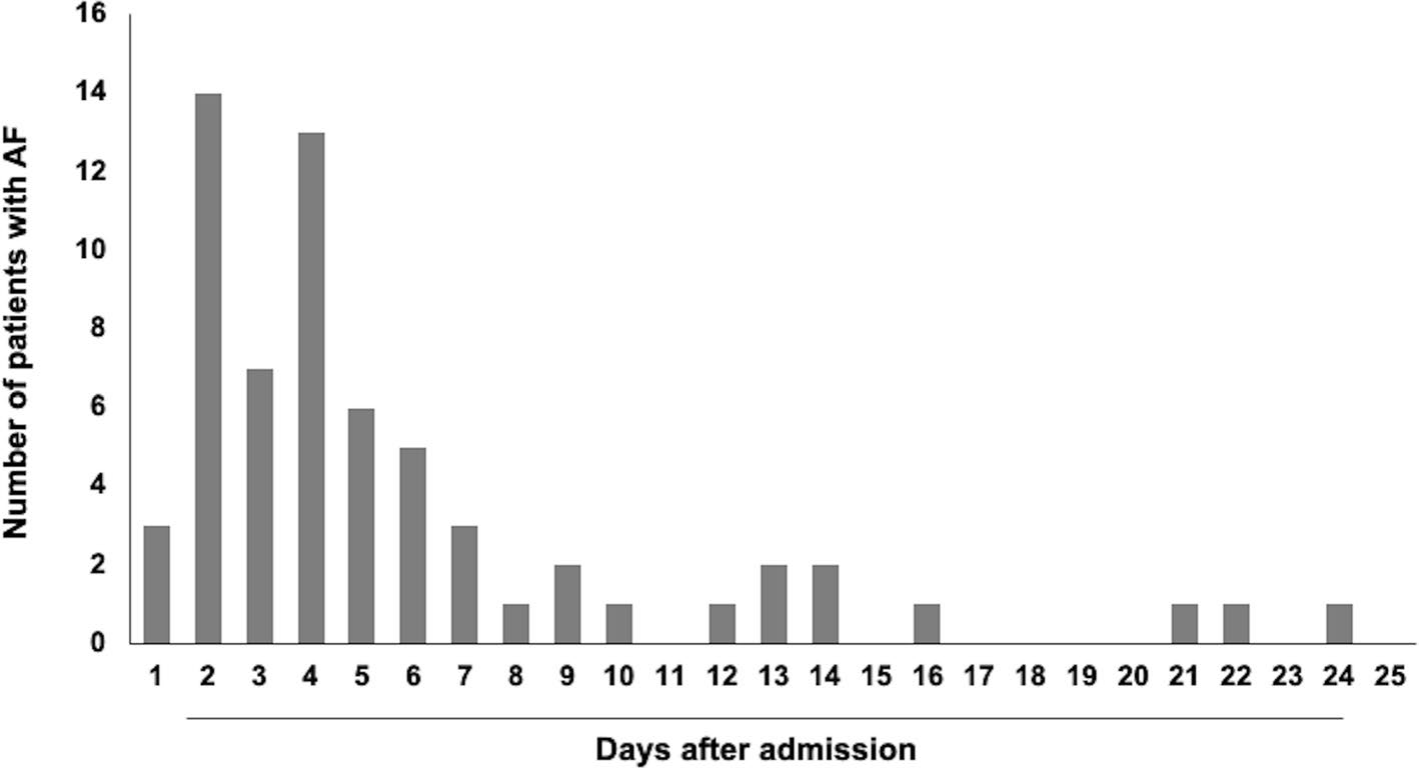
Temporal profile of atrial fibrillation development after admission. Histograms show the number of patients with detection of atrial fibrillation according to the day after admission.

A total of 677 patients with cryptogenic strokes (mean age, 68.7 ± 12.8 years; 455 males) were enrolled in the present study. The median baseline National Institutes of Health Stroke Scale (NIHSS) score was 2, and AF was detected in 64 patients (9.5%). The median length of hospital stay was 17 days. The temporal profile of AF detection during hospitalization is shown in Fig. 1 among patients with detection. Four days after admission was identified as the approximate median day for detection of AF and was used to classify early and late AF detection. Based on this, AF was found in ≤ 4 days in 37 patients and in > 4 days in 27 patients.

### Clinical characterizations according to early, late, and no detection of AF in cryptogenic stroke

Baseline characteristics of the entire study population, cryptogenic stroke patients with in-hospital early and late AF development, and absence of AF detection are summarized in Table 1. The mean age of patients was in the following order: early AF development, late AF development, and absence of AF (77.6 ± 7.4, 75.6 ± 10.6, 67.9 ± 12.8 years, respectively, *p* < 0.001). The female sex and CKD were more predominant in patients with early AF detection than in other groups (49%, *p* = 0.034; 57%, *p* = 0.039), whereas the frequency of cigarette smoking was lower in patients with early AF development (3%, *p* < 0.001). The median NIHSS score was 5 in patients with early AF development, which was higher than in other groups (*p* < 0.001). Functional outcomes, based on the modified Rankin Scale, at discharge were not different among the groups. Findings from magnetic resonance imaging showed that a large infarction (> 3 cm in diameter) was significantly more common in patients with late AF development than in patients with early AF development and without AF (59%, *p* = 0.001). In echocardiographic parameters, left atrial (LA) diameter was larger in patients with early and late AF development (38.9 ± 5.9, 38.6 ± 5.0 mm, respectively, *p* < 0.001) than in those without AF. In comparison, LAA flow was lower in patients with early and late AF development than in patients without AF (48.1 ± 16.0, 47.5 ± 14.7 cm/s, respectively, *p* < 0.001). The SEC frequency observed in the patients was in the following order: early AF development, late AF development, and without AF (32%, 19%, and 3%, respectively, *p* < 0.001). Aortic arch plaques on TEE were more frequent in patients without AF detection (39%, *p* = 0.018). In laboratory data, brain natriuretic peptide (BNP) levels of patients were in the following order: early AF development, late AF development, and without AF (175.5 ± 131.3, 134.1 ± 116.5, 94.3 ± 166.9 pg/mL, respectively, *p* < 0.001).

**Table 1:** Baseline characteristics, MRI and echocardiographic findings, and laboratory data in AF development ≤ 4 days and > 4 days, and none in the CHALLENGE ESUS/CS study population.

Characteristics	Atrial fibrillation			*p*
	≤ 4 days	> 4 days	Negative	
	n = 37, 5%	n = 27, 4%	n = 613, 91%	
**Sociodemographic**				
Age, years, mean ± SD	77.6 ± 7.4	75.6 ± 10.6	67.9 ± 12.8	< 0.001
Gender, female, no (%)	18 (49)	5 (19)	199 (32)	0.034
**Risk factors, no (%)**				
Hypertension	31 (84)	20 (74)	433 (71)	0.217
Diabetes Mellitus	5 (14)	6 (22)	161 (26)	0.208
Dyslipidemia	18 (49)	13 (48)	314 (51)	0.913
Cigarette smoking	1 (3)	7 (26)	173 (28)	< 0.001
Coronary artery disease	3 (8)	5 (19)	60 (10)	0.310
Chronic kidney disease	21 (57)	9 (33)	222 (36)	0.039
Previous history of ischemic stroke	3 (8)	2 (7)	118 (19)	0.052
NIHSS score on admission, median (IQR)	5 (3–10)	3 (2–9)	2 (1–5)	< 0.001
Modified Rankin Scale at discharge, median (IQR)	1 (0–3)	1 (0–2)	1 (0–2)	0.853
CHADS 2 score, median (IQR)	2 (1–2)	2 (1–2)	2 (1–2)	0.493
CHADS2-VASc score, median (IQR)	3 (2–4)	3 (2–4)	3 (2–4)	0.121
**MRI, no (%)**				
Large infarction > 3 cm in diameter^a^	15 (41)	16 (59)	170 (28)	0.001
Multiple infarction^a^	30 (81)	18 (67)	383 (62)	0.080
Cortical infarction^a^	32 (86)	25 (93)	482 (79)	0.108
**Echocardiographic findings**				
LA diameter, cm/s, mean ± SD^b^	38.9 ± 5.9	38.6 ± 5.0	35.0 ± 6.4	< 0.001
LAA flow, cm/s, mean ± SD^c^	48.1 ± 16.0	47.5 ± 14.7	57.6 ± 17.5	< 0.001
Ejection fraction, %	64.7 ± 7.0	62.9 ± 5.3	63.8 ± 8.1	0.539
Spontaneous echo contrast, no (%)	12 (32)	5 (19)	17 (3)	< 0.001
ASA^d^	2 (5)	3 (11)	87 (14)	0.229
RLS^e^	9 (36)	8 (32)	295 (49)	0.125
Aortic arch plaques^f^	6 (16)	9 (33)	239 (39)	0.018
**Laboratory findings**				
BNP, pg/ml, mean ± SD^g^	175.5 ± 131.3	134.1 ± 116.5	94.3 ± 166.9	< 0.001
D-dimer, μg/mL, mean ± SD	1.4 ± 0.9	1.4 ± 0.9	3.1 ± 17.1	0.851

Chi-square test, and the Kruskal–Wallis test were used for comparison.

AF = atrial fibrillation; NIHSS = NIH Stroke scale; IQR = interquartile range; LA = left atrium; LAA = left atrial appendage; ASA = atrial septal aneurysm; RLS = right-to-left shunt; BNP = brain natriuretic peptide.

Missing values: ^a^n = 5; ^b^n = 44; ^c^n = 25; ^d^n = 6; ^e^n = 23; ^f^n = 2; ^g^n = 94. Chronic kidney disease was defined as eGFR < 60 mL/min/1.73 m^2^. LAA flow was defined an average of LAA inflow and out flow.

### Independent variables associated with early and late detection of paroxysmal AF (PAF)

Age, female sex, cigarette smoking, CKD, NIHSS score, large infarct > 3 cm, LA diameter, LAA flow, SEC, aortic arch plaques, and BNP were entered into multinomial logistic regression analyses. Age (odds ratio [OR] 1.06; 95% confidence interval [CI] 1.01–1.11; *p* = 0.019), LA diameter (OR 1.07; 95% CI 1.01–1.14; *p* = 0.036), and SEC (OR 5.91; 95% CI 2.19–15.97; *p* < 0.001) were associated with early detection of AF, whereas cigarette smoking (OR 0.12; 95% CI 0.02–0.92; *p* = 0.042) and aortic arch plaques (OR 0.21; 95% CI 0.08–0.61; *p* = 0.004) were inversely related (Table 2). Age (OR 1.07; 95% CI 1.01–1.12; *p* = 0.012) and a large infarction > 3 cm in diameter (OR 3.28; 95% CI 1.35–7.97; *p* = 0.009) were associated with late detection of AF (Table 2).

**Table 2:** Multinomial logistic regression analysis predicting factors associated with AF development ≤ 4 days and > 4 days.

Variables	Detection of AF < 4 days			Detection of AF > 4 days		
	OR	95% CI	*p*	OR	95% CI	*p*
Age	1.06	1.01–1.11	0.019	1.07	1.01–1.12	0.012
Female	0.93	0.40–2.17	0.871	0.30	0.10–0.92	0.035
Cigarette smoking	0.12	0.02–0.92	0.042	1.07	0.40–2.87	0.897
Chronic kidney disease	1.42	0.63–3.17	0.395	0.61	0.24–1.54	0.295
NIHSS score on admission	1.04	0.99–1.10	0.119	1.02	0.95–1.08	0.643
Large infarction ≥ 3 cm in diameter	1.18	0.50–2.77	0.707	3.28	1.35–7.97	0.009
LA diameter	1.07	1.01–1.14	0.036	1.05	0.98–1.13	0.169
LAA flow	0.99	0.97–1.02	0.668	0.97	0.95–1.00	0.078
SEC	5.91	2.19–15.97	< 0.001	2.97	0.82–10.82	0.099
Aortic arch plaques	0.21	0.08–0.61	0.004	0.53	0.21–1.31	0.167
BNP	1.00	1.000–1.004	0.081	1.00	0.998–1.003	0.749

LAA flow was defined an average of LAA inflow and out flow.

AF = atrial fibrillation; NIHSS = NIH Stroke scale; LA = left atrium; LAA = left atrial appendage; SEC = spontaneous echocontrast; BNP = brain natriuretic peptide.

## Discussion

The current study elucidated the factors related to in-hospital detection of AF in cryptogenic stroke, depending on the early and late phases of hospitalization using data from the CHALLENGE ESUS/CS registry. The principal findings are that AF was detected during hospitalization in 9.5% of cryptogenic stroke patients, and using 4 days as the approximate cutoff after admission, SEC and large infarct size > 3 cm in diameter were found to be significantly related to AF development in the early and late phases, respectively.

Detection of covert AF is critical in cryptogenic stroke and ESUS patients. Although our data did not show a significant difference in functional outcomes at discharge in patients with early and late AF development, and absence of AF detection, covert AF has been shown to increase the risk of a recurrent stroke long-term among patients with embolic diseases^19^. Many previous studies have focused on the factors that predicted covert AF in cryptogenic stroke and ESUS. Electrophysiological substrates, such as P wave alterations and supraventricular extrasystole^20,21^, and blood biomarkers, such as BNP and NT-proBNP^22,23^, could predict occult AF in cryptogenic stroke and ESUS. Importantly, LA enlargement, SEC, and LAA dysfunction were related to not only covert AF in cryptogenic stroke and ESUS^24,25^, but also to embolic events in patients with AF^14–16^. In the sub-analysis of NAVIGATE ESUS, patients with an LA diameter > 46 mm displayed lower stroke recurrence after rivaroxaban treatment^26^. Thus, echocardiographic parameters are important to predict AF in cryptogenic stroke and ESUS. On the other hand, some studies have explored the clinical significance of early and late development of AF. After catheter ablation, early and late recurrences of AF showed different clinical characteristics, and an early recurrence of AF ≤ 7 days predicted a late recurrence of AF^10,12^. In acute myocardial infarctions, comorbidity with AF occurring in the late phase of > 24 h, but not AF resolved within ≤ 24 h, displayed a higher incidence of stroke rate compared to no AF^11^. In our data, increasing age was related to both early and late AF detection, which was consistent with previous studies^2,27^. Conversely, current smoking habits were shown to be negatively associated with early detection of PAF. In a previous study, current smoking status was more closely related to stroke patients aged < 65 years than to those aged ≥ 75 years^28^. Thus, it is suggested that older patients did not have smoking habits, reflecting the current results of the association between aging and AF detection. More importantly, among various important characterizations related to detection in early and late stages with a cutoff of 4 days, SEC and large infarction were closely linked with the early and late occurrence of AF, respectively.

In recent years, there has been growing interest in the detection of AF using prolonged cardiac monitoring using ICM. A recent meta-analysis identified that the detection rates of AF with ICM were 5%, 21%, 26%, and 34% in < 6 months, 6–12 months, 12–24 months, and ≥ 24 months, respectively; increasing age was correlated with AF detection, whereas sex, atherosclerotic risk factors, CHADS2 score, and CHA2DS2-VASc score were not^29^. Meanwhile, AF was detected in a small number of patients with ESUS in large-scale clinical trials where systematic assessment for AF by ICM was not carried out^8,9^. Therefore, there is a possibility that AF detection by ICM may ensure the efficacy of direct oral anticoagulants in patients with cryptogenic stroke or ESUS. In our data, SEC was closely related to the early detection of AF and was also relatively linked with the late development of AF, whereas a large infarction was an independent factor for the late development of AF in cryptogenic stroke. So far, clinical characteristics related to long-term AF detection are not well understood. Furthermore, it was shown that SEC was related to AF^30^, while the association of radiological parameters such as large infarctions, shown in the current study, with long-term AF detection on ICM are yet to be elucidated. Thus, not only SEC, but also large infarctions could be important characteristics for an ICM recommendation, even though AF was not detected during hospitalization in those patients; further studies are warranted.

This study has some limitations when interpreting the present results. First, this was a retrospective study with a small sample size of early and late detection of AF. Furthermore, the period of continuous cardiac monitoring and the timing of a 24-h Holter electrocardiogram differed among individuals and were determined at the discretion of the treating physicians; this raised a potential bias for AF detection. In addition, unknown stroke patients with detection of AF < 24 h were diagnosed with cardioembolic stroke and not registered in the CHALLENGE ESUS/CS database. Second, selection bias may have been involved in the performance of TEE in patients with cryptogenic stroke at participating hospitals. Third, radiological, echocardiographic, and laboratory data were lacking in a fraction of cases.

## Conclusion

The CHALLENGE ESUS/CS registry enrolled a large number of cryptogenic stroke patients with comprehensive data. Our results showed that SEC and large infarct size were important predictors of AF detection, particularly in the early and late phases, respectively. Clinical characteristics including radiological parameters related to long-term AF detection are not well understood. The presence of SEC and a large infarction could be indications for recommending ICM.

## Supplementary Information

Supplementary Information
