# Supplementary material for: Different aspects of early and late development of atrial fibrillation during hospitalization in cryptogenic stroke
Source: Sci Rep. 2021 Mar 29;11:7127. doi: 10.1038/s41598-021-86620-5 (PMC8007744; doi:10.1038/s41598-021-86620-5)
Supplement: Supplementary file 1 — Supplementary Information [file 41598_2021_86620_MOESM1_ESM.pdf]

# Different aspects of early and late development of atrial fibrillation during hospitalization in cryptogenic stroke

Ryosuke Doijiri, MD[1)]; Yuji Ueno, MD, PhD[2)*]; Muneaki Kikuno, MD[3,4)]; Takahiro Shimizu, MD, PhD[5)]; Yohei Tateishi, MD, PhD[6)]; Ayako Kuriki, MD, PhD[7)]; Hidehiro Takekawa MD, PhD[8)]; Yoshiaki Shimada, MD, PhD[9)]; Kodai Kanemaru, MD[3,4)]; Yuki Kamiya, MD, PhD[7)]; Eriko Yamaguchi, MD[1)]; Masatoshi Koga, MD, PhD[3)]; Masafumi Ihara, MD, PhD[10)]; Akira Tsujino, MD, PhD[6)]; Koichi Hirata, MD, PhD[8)]; Yasuhiro Hasegawa, MD, PhD[5)]; Takahiko Kikuchi, MD, PhD[1)], Nobutaka Hattori, MD, PhD[2)]; Takao Urabe, MD, PhD[9)]

1) Department of Neurology, Iwate Prefectural Central Hospital, Iwate, Japan
2) Department of Neurology, Juntendo University Faculty of Medicine, Tokyo, Japan
3) Department of Cerebrovascular Medicine, National Cerebral and Cardiovascular Center, Osaka, Japan
4) Department of Neurology, Tokyo Medical School, Tokyo, Japan
5) Department of Neurology, St. Marianna University School of Medicine, Kanagawa, Japan
6) Department of Neurology and Strokology, Nagasaki University Hospital, Nagasaki, Japan
7) Department of Neurology, Showa University Koto Toyosu Hospital, Tokyo, Japan
8) Department of Neurology, Dokkyo Medical University, Tochigi, Japan
9) Department of Neurology, Juntendo University Urayasu Hospital, Chiba, Japan
10) Department of Neurology, National Cerebral and Cardiovascular Center, Osaka, Japan

**CHALLENGE ESUS/C.S. collaborators**

**Department of Neurology, Juntendo University Faculty of Medicine, Tokyo, Japan:** Yuji Ueno, Nobutaka Hattori, Kenichiro Hira.

**Department of Neurology, Iwate Prefectural Central Hospital, Iwate, Japan:** Ryosuke Doijiri, Eriko Yamaguchi, Takahiko Kikuchi.

**Department of Neurology, Juntendo University Urayasu Hospital, Chiba, Japan:** Yoshiaki Shimada, Takao Urabe, Naohide Kurita.

**Department of Neurology, Showa University Koto Toyosu Hospital, Tokyo, Japan:** Ayako Kuriki, Yuki Kamiya.

**Department of Neurology and Strokology, Nagasaki University Hospital, Nagasaki, Japan:** Yohei Tateishi, Akira Tsujino, Tadashi Kanamoto.

**Department of Neurology, St. Marianna University School of Medicine, Kanagawa, Japan:** Takahiro Shimizu, Yasuhiro Hasegawa, Yuta Hagiwara.

**Department of Neurology, Dokkyo Medical University, Tochigi, Japan:** Hidehiro Takekawa, Koichi Hirata, Ayano Suzuki.

**Department of Cerebrovascular Medicine, National Cerebral and Cardiovascular Center, Osaka, Japan:** Muneaki Kikuno, Kodai Kanemaru, Masatoshi Koga.

**Department of Neurology, National Cerebral and Cardiovascular Center, Osaka, Japan:** Masafumi Ihara.

**Department of Neurology, Tokyo Medical School, Tokyo, Japan:** Muneaki Kikuno, Kodai Kanemaru.
